# Supplementary material for: Positive and negative regulation of carbon nanotube catalysts through encapsulation within macrocycles
Source: Nat Commun. 2018 Jul 10;9:2671. doi: 10.1038/s41467-018-05183-8 (PMC6039438; doi:10.1038/s41467-018-05183-8)
Supplement: Supplementary file 1 — Supplementary Information [file 41467_2018_5183_MOESM1_ESM.pdf]

SUPPLEMENTARY INFORMATION

Positive and negative regulation of carbon nanotube catalysts through encapsulation within macrocycles

Matías Blanco,[1] Belén Nieto-Ortega,[1] Alberto de Juan,[1] Mariano Vera-Hidalgo,[1] Alejandro López-Moreno,[1] Santiago Casado,[1] Luisa R. González,[2] Hidetaka Sawada,[3] José M. González-Calbet,[2] and Emilio M. Pérez.[1,*]

1) IMDEA Nanociencia, Ciudad Universitaria de Cantoblanco, c/Faraday 9, 28049 Madrid, Spain.

E-mail: emilio.perez@imdea.org

2) Departamento de Química Inorgánica, Universidad Complutense de Madrid, 28040, Madrid, Spain.

3) JEOL Ltd, 3-1-2 Musashino, Akishima, Tokyo, 196-8558, Japan.

Supplementary Methods

Materials: (6,5)-Enriched single walled nanotubes (6,5-SWNT) were purchased from Sigma-Aldrich (0.7−0.9 nm in diameter, length ≥700 nm, mostly semiconducting, 95% purity). Reagents were used as purchased. All solvents were dried according to standard procedures. All air-sensitive reactions were carried out under N_2 atmosphere.

Characterization methods: Analytical thin layer chromatographies (TLC) were performed using aluminium-coated Merck Kieselgel 60 F254 plates. NMR spectra were recorded on a Bruker Avance 400 (^{1}H: 400 MHz; ^{13}C: 100 MHz) spectrometers at 298 K, using partially deuterated solvents as internal standards. Coupling constants (*J*) are denoted in Hz and chemical shifts (δ) in ppm. Electrospray ionization mass spectrometry (ESI-MS) and Matrix-assisted Laser desorption ionization (coupled to a Time-Of-Flight analyzer) experiments (MALDITOF) were recorded on a HP1100MSD spectrometer and a Bruker REFLEX. Thermogravimetric analyses (TGA) were performed using a TA Instruments TGAQ500 with a ramp of 10 °C min^{-1} under air from 100 to 1000 °C. Transmission electron microscopy (TEM) images were obtained with JEOL-JEM 2100F instrument or a JEOL-JEM GRAND ARM300cF (AC-HRTEM). Atomic Force Microscopy (AFM) images were acquired using a JPK NanoWizard II AFM working in dynamic mode. NT-MDT NSG01 silicon cantilevers, with typical values of 5.1 N m^{-1} spring constant and 150 kHz resonant frequency, were employed under ambient conditions in air. Total reflection X Ray Fluorescence analyses (TRXF) were performed on a TXRF 8030c - FEI Spectrometer. Raman spectra were acquired with a Bruker Senterra confocal Raman microscope instrument equipped with 532, 633 and 785 nm excitation lasers. UV-vis-NIR spectra were performed using a Shimadzu

UV-VIS-NIR Spectrophotometer UV-3600. Photoluminescence excitation intensity maps (PLE) were obtained with NanoLog 4 HORIBA instrument.

Synthesis and characterization of macrocycle mac-AQ: A catalytic amount of Grubb's 1st generation catalyst was added to a solution of the corresponding linear precursor[1] in dry and degassed DCM, and the mixture was stirred at room temperature. The progress of the reaction was monitored by TLC. When the starting linear precursor was consumed, the reaction was stopped by filtration through a pad of celite. Solvent was evaporated under reduced pressure, and the crude was purified by flash chromatography (Hex:AcOEt 3:1) to obtain the product in 70% yield.

^{1}H NMR (400 MHz, $CDCl_3$, 298 K) δ 7.93 – 7.89 (m, 4H, Hi), 7.40 – 7.28 (m, 8H, 4Hj+4Hl), 7.03 – 6.99 (m, 4H, Hh), 5.39 (t, *J* = 3.9 Hz, 2H, Ha), 5.33 (s, 4H, Hk), 4.07 (t, *J* = 6.8 Hz, 4H, Hg), 2.07 (dd, *J* = 15.6, 5.9 Hz, 4H, Hf), 1.87 – 1.80 (m, 4H, Hb), 1.49 – 1.38 (m, 12H, Hc-He).^{13}C NMR (101 MHz, $CDCl_3$) δ 181.7, 181.2, 163.8, 162.6, 137.2, 135.5, 135.4, 131.1, 129.5, 127.1, 126.7, 126.0, 124.3, 121.1, 120.5, 111.8, 110.8, 69.6, 68.7, 32.0, 29.4, 29.0, 28.1, 25.7. HRMS-MALDI calculated for $C_{50}H_{46}NaO_8$ $[M+Na]^+$: 797.3085; found 797.3114.

Synthesis and characterization of MINTs: The general method for the synthesis of MINTs has been reported elsewhere[2,3]. Briefly, the nanotubes (10 mg) were suspended in 10 mL of tetrachloroethane through sonication (10 min) and mixed with 0.01 mmol of linear bisalkene U-shaped precursors of the macrocycles mac-exTTF, mac-pyr or mac-AQ, and Grubbs' second-generation catalyst at room temperature for 72 h. After this time, the suspension was filtered through a PTFE membrane of 0.2 μm pore size and the solid washed profusely with DCM. The solid was resuspended in 10 mL of DCM through sonication for 10 min and filtered through a PTFE membrane of 0.2 μm

pore size again. This washing procedure was repeated three times. Samples obtained were denoted as **MINT-exTTF**, **MINT-pyr** or **MINT-AQ** as a function of the threading macrocycle around the nanotubes. The synthesis of the supramolecular complexes denoted as **SWNT-exTTF**, **SWNT-pyr** and **SWNT-AQ** was performed by the direct mixing of the adequate amounts of 6,5-SWNT and the corresponding macrocycle without catalyst to achieve the same functionalization loading of organic material over the nanotube compared to their respective MINT sample.

DFT calculations: All theoretical DFT calculations were carried out within the density functional theory (DFT) approach by using the C.01 revision of the Gaussian 09 program package.[4] Optimization and molecular orbitals calculations of MINT derivative were performed using the long-range corrected B97D density functional[5], which are able to incorporate the dispersion effects by means of a pair-wise London-type potential. The B97D density functional has emerged as a robust and powerful density functional able to provide accurate structures in large supramolecular aggregates dominated by non-covalent interactions of different nature. Raman spectra and analysis of Mülliken population were simulated by using the Coulomb-attenuated hybrid exchange-correlation functional (CAM-B3LYP) functional. This functional was develop by Yanai et al[6]. which includes the Hartree-Fock and the Becke exchanges as a variable ratio depending of the intermolecular distance. Both functional were combined with the Pople's 3-21G* basis set[7]. The dimension of the **MINT-exTTF** **(**Supplementary Figure 9) is an example of the size of our systems.

Binding energies: The interaction energy between the macrocycles or the substrates (E_{int}) (Supplementary Equation 1) and the carbon nanotube (Supplementary Table 1, 2) and **MINT-AQ** (Supplementary Table 3) is defined as the energy difference between the two fully optimized monomers from the fully optimized dimer complex in the

geometry of the dimer complex, where E_x^y is the energy of fragment X at the geometry of Y. The basis set superposition error (BSSE) was half-corrected according to the counterpoise (CP) scheme of Boyd and Bernardi for the single-point interaction energies.[8] Otherwise, the binding energy (E_{bind}) (Supplementary Equation 2) was calculated taking into account the relaxation of the separate monomers and, therefore, considering the deformation energy required to transform the both moieties from their minimum-energy geometries to the geometry acquired in the assembly.

Mülliken population: The analysis of Mülliken population was carried out at CAM-B3LYP/3-21g* level of theory (Supplementary Table 4). An extra example at CAM-B3LYP/6-31g* level for the case of MINT-AQ has been included to support that a change in the basis set does not modify the sense of the charge transfer.

Catalytic activity: The typical catalytic experiment was performed as follows: a certain amount of nanotube-containing material (5 mg) was mixed with 4.1 mmol of nitroaromatic substrate in 2 mL of d_6-DMSO as solvent (selected after reaction condition optimizations, Supplementary Figure 13) in a round botton flask under N_2 atmosphere. Finally, 31.8 mmol of hydrazine (acting as hydrogen source[9]) were added and the reaction was stirred magnetically and held at 85 ºC for a desired time. At regular intervals, aliquots were withdrawn from the reaction and subjected to NMR spectroscopy analysis to follow the catalytic evolution (Supplementary Figures 19-25).

Once the reaction was complete, the crude mixture was diluted with 15 mL of DCM and the MINT-containing solid catalysts **MINT-exTTF**, **MINT-pyr** and **MINT-AQ** were recovered by filtration through a PTFE membrane of 0.2 μm pore-size and washed profusely with DCM. The solid was re-suspended in 10 mL of DCM through sonication for 10 min and filtered through a PTFE membrane of 0.2 μm pore size again. This

washing procedure was repeated three times. After drying, the material was submitted to another catalytic run without adding in any case new catalyst precursor.

To isolate the pure products, the organic phase was washed three times with water. Then, organic fractions were dried over anhydrous $MgSO_4$ and concentrated under reduced pressure. Crude product was purified by flash chromatography in silica (hexane:ethyl acetate 3:1) yielding the final product.

Analysis of X-Ray Fluorescence (TRXF): In order to confirm that the active sites corresponded only to the nanotube walls, we conducted total reflected X-ray fluorescence (TRXF) measurements, and only ppm-level of metallic impurities were detected (Supplementary Figures 15, 16, 17 and 18).

Characterization of the products obtained in the nitroarene reduction

Aniline.

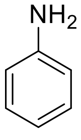

Prepared according to the general procedure. ^{1}H-NMR (400.16 MHz, DMSO-d_6, δ ppm): 7.02 (t, J = 7.3 Hz, 2H), 6.56 (d, J = 7.2 Hz, 1H), 6.49 (t, J = 7.3 Hz 2H), 4.99 (s, 2H).

1-aminonaphthalene.

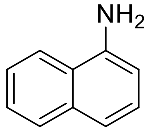

Prepared according to the general procedure. ^{1}H-NMR (400.16 MHz, DMSO-d_6, δ ppm): 8.05 (d, J = 8.3 Hz, 1H), 7.73 (d, J = 8.2 Hz, 1H), 7.37 (m, 2H), 7.19 (t, J = 7.7 Hz, 2H), 7.07 (d, J = 8.0 Hz, 1H), 6.67 (d, J = 7.3 Hz 2H), 5.68 (s, 2H).

9-aminoanthracene.

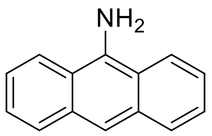

Prepared according to the general procedure. ^{1}H-NMR (400.16 MHz, DMSO-d_6, δ ppm): 8.59 (s, 1H), 8.13 – 8.06 (m, 4H), 7.53 (dd, J = 6.6, 3.2 Hz, 4H), 6.87 (s, 2H).

1-Aminopyrene.

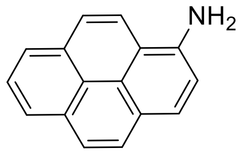

Prepared according to the general procedure. ^{1}H-NMR (400.16 MHz, DMSO-d_6, δ ppm): 8.26 (d, J = 9.2 Hz, 1H), 8.02 – 7.84 (m, 6H), 7.72 (d, J = 8.8 Hz, 1H), 7.36 (d, J = 8.3 Hz, 1H), 6.32 (s, 2H).

Supplementary Discussion

Analysis of the MINT-forming reaction: We have previously observed that under the MINT-forming reaction conditions, besides MINTs, oligomers of the bisalkene macrocycle precursors can be formed in-situ through acyclic diene metathesis polymerization (ADMP) and wrap around SWNTs forming supramolecular associates that are stable enough to survive our purification process[10]. To unambigously discard a significant participation of oligomer-wrapped SWNTs in our final MINT product we analysed the kinetics of formation of **MINT-AQ**. To that end, we extracted aliquots at different reaction times and performed TGA of the products after purification. If Ring-Closing Metathesis (RCM) is the rate-determining step, the kinetics of the reaction must be of pseudo first order and the final products are MINTs. However, if ADMP were the main reaction pathway, the kinetics would correspond to a bimolecular reaction and follow second order kinetics.

The data from the formation of **MINT-AQ** fit very well (r^2 = 0.988) to a pseudo-first order model with kinetic constant of 2.2×10^{-3} s^{-1}. Therefore, the main path for functionalization of SWNTs is by macrocyclization around them, to form MINTs, with

negligible contribution from supramolecularly attached oligomers (Supplementary Figure 8). For comparison, we also studied the use of fullerene C_{60} as soluble template for the RCM, since its diameter (0.7 nm) is very similar to that of (6,5)-SWNTs. To follow the reaction in real time by ^{1}H-NMR, we performed the experiment using 1,1,2,2-tetrachloroethane-d_2 as solvent and recorded spectra at different times. As the reaction progresses, the signal at 4.19 ppm that corresponds to the $-CH_2$ group next to the oxygen ether group in the bisalkene precursor decreases and a new signal a 4.13 ppm, which corresponds to the macrocycle, appears. The data obtained from this experiment also fit well (r^2 = 0.982) to a pseudo-first order reaction kinetics, in this case with $k = 5.6 \pm 0.5 \times 10^{-4}\ s^{-1}$. Since in this case we can unambiguously identify mac-AQ as the sole product of the reaction detectable by NMR, these data strongly support the formation of MINTs by analogy. Moreover, (6,5)-SWNTs are found better templates for the RCM than C_{60}, as the RCM reaction is approximately twice as fast with the nanotubes as templates.

Mülliken population: An analysis of Mülliken population suggests that there is charge-transfer between the macrocycles and SWNT. For MINT-AQ, the extent of electron transfer upon the formation of the MINT in the SWNT is found to be +0.011 e (p-doping), while for the formation of the MINT-exTTF the value is -0.043 e (n-doping), higher and with opposite sign. This is consistent with the nature of electron-withdrawing tendency of the AQ and electron-donor tendency of exTTF. For the MINT-pyr, the calculated charge transfer is +0.005 e, one order the magnitude lower and it is not a charge transference between the two moieties. These theoretical results are in relay

good agreement with the Raman experimental tendencies (Fig. 2b in the main text) and with our interpretation of the catalysis data.

Raman Calculations: Raman spectra of **MINT-exTTF, MINT-pyr** and **MINT-AQ** along with the Raman spectrum of pristine (6,5)-SWNT (Supplementary Figure 10a). All spectra are very similar, proving that the covalent structure of the SWNT is preserved upon formation of MINTs, with no increase in the relative intensity of the D band. However, the intensity of the radial breathing modes of all three MINT samples decreases with respect to (6,5)-SWNT, in accordance with the type of functionalization[11]. Some variations were observed in the frequency of the G band, too. For instance, in the cases of **MINT-exTTF** and **MINT-AQ** we observe a small blue shift of 2-5 cm^{-1}, but with **MINT-pyr** we do not observe remarkable variations. The fact that both the electron donor mac-exTTF and acceptor mac-AQ cause blue shifts[12] and that these are quantitatively small, suggests that the degree of charge-transfer in the ground state is small.

To shed light on the underlying causes of these spectroscopic changes, we performed DFT calculations at the CAM-B3LYP/3-21g* level of theory (Figure 1B of the main text). All three macrocycles are a good fit for (6,5)-SWNTs and show sizeable binding energies towards them (−103.58, −108.61 and −97.74 Kcal mol^{-1} for **MINT-exTTF**, **MINT-Pyr** and **MINT-AQ**, respectively). The calculated Raman spectra (Supplementary Figure 10b) showed the same blue shift of 5 cm^{-1} upon formation of **MINT-exTTF** and **MINT-AQ**, in very good correlation with our experimental results. However, we also observe a blue shift for the **MINT-pyr** that we do not see experimentally.

HOMO-LOMO calculations: The energy gap between the highest occupied molecular orbital (HOMO) and the lowest unoccupied molecular orbital (LUMO) plays an

important role in the charge transport properties of any organic molecule (Supplementary Figure 11). Specifically, in semiconducting SWNTs the HOMO-LUMO gap corresponds to absorption or luminescence of the lowest-energy transition (S_{11}). The calculations show that the degenerated HOMO/HOMO-1 of **MINT-exTTF** is mainly formed from a combination of the molecular orbitals (MOs) of the macrocycle threaded along the nanotube and the carbon scaffold, whereas its LUMO/LUMO+1 is formed only by **6,5-SWNT** MOs (Supplementary Figure 11). The opposite trend is observed for **MINT-AQ**, where the HOMO/HOMO-1 reside exclusively on the SWNT, while the LUMO/LUMO+1 are located on mac-AQ. Interestingly, for **MINT-pyr,** both HOMO and LUMO are located on the SWNT, without any participation of mac-pyr. These changes in the nature of MOs confirm the different electronic characteristics of each MINT sample. The HOMO of **MINT-exTTF** is significantly higher in energy than that of **MINT-pyr**, in correspondence with the donor behavior of mac-exTTF compared to mac-pyr. On the other hand, the LUMO of **MINT-AQ** is lower in energy than the corresponding LUMO of **MINT-pyr**, as is expected for an acceptor. The energy gap between the HOMO and the LUMO ($\Delta E_{HOMO\text{-}LUMO}$) can be correlated with the conductivity of the complex. Experimentally, by spectrofluorimetric measurements, (6,5)-SWNTs have a $\Delta E_{HOMO\text{-}LUMO}$ of 1.27 eV[13], in good correlation with our calculated value of 1.22 eV, which support our theoretical data. This value decreases to 0.63 eV for **MINT-exTTF**, while is increased to 1.29 eV and 1.26 eV for **MINT-pyr** and **MINT-AQ** respectively (Supplementary Figure 11). For **MINT-exTTF**, the first orbital with electronic density that resides mainly on the carbon nanotube moiety is found at 4.29 eV in the HOMO-4, at a significantly higher value that for **MINT-pyr** and **MINT-AQ**. All these theoretical results, together with the experimental findings, indicate that the

electronic character of our MINT samples can be modulated changing the recognition motifs of U-shape precursors.

<u>*Catalytic activity:*</u> The evolution of the ^{1}H-NMR spectra during the reduction of Ph-NO_2 with **MINT-exTTF** as catalyst (Supplementary Figure 14a) proceeds smoothly with no induction period detected, yielding the aniline reduction product almost exclusively, with phenylhydroxylamine as only detectable intermediate. The transformation can be easily monitored by the disappearance of the up-shielded protons of the starting material at ~ δ 8.25-7.75 ppm and the increase of the signals down-shielded at ~ δ 6.75–6.25 ppm as a consequence of the increase in electron density of the aromatic ring caused by the aniline nitrogen, and the characteristic 2-proton singlet of the aniline group at ~ δ 5.0 ppm. The reduction proceeds to completion in approximately 2 hours. Compared to a blank non-catalyzed reaction, which saturated after 24 h with ~50% of conversion (Supplementary Figure 14b), and resulted in an approximately equimolar mixture of phenylhydroxylamine and aniline, **MINT-exTTF** showed remarkably activity.

Supplementary Figures

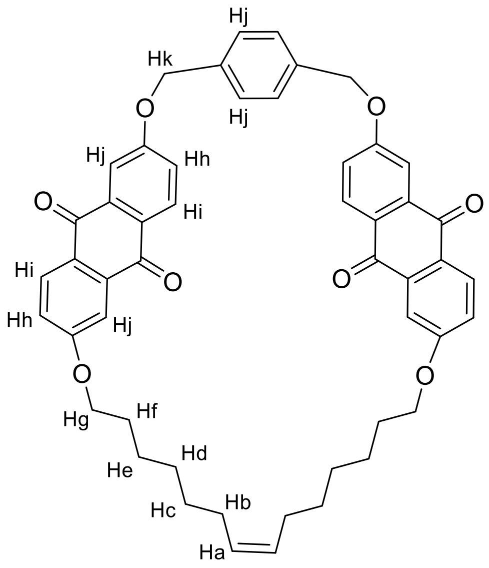

Supplementary Figure 1. Proposed structure of **mac-AQ** with the assignation.

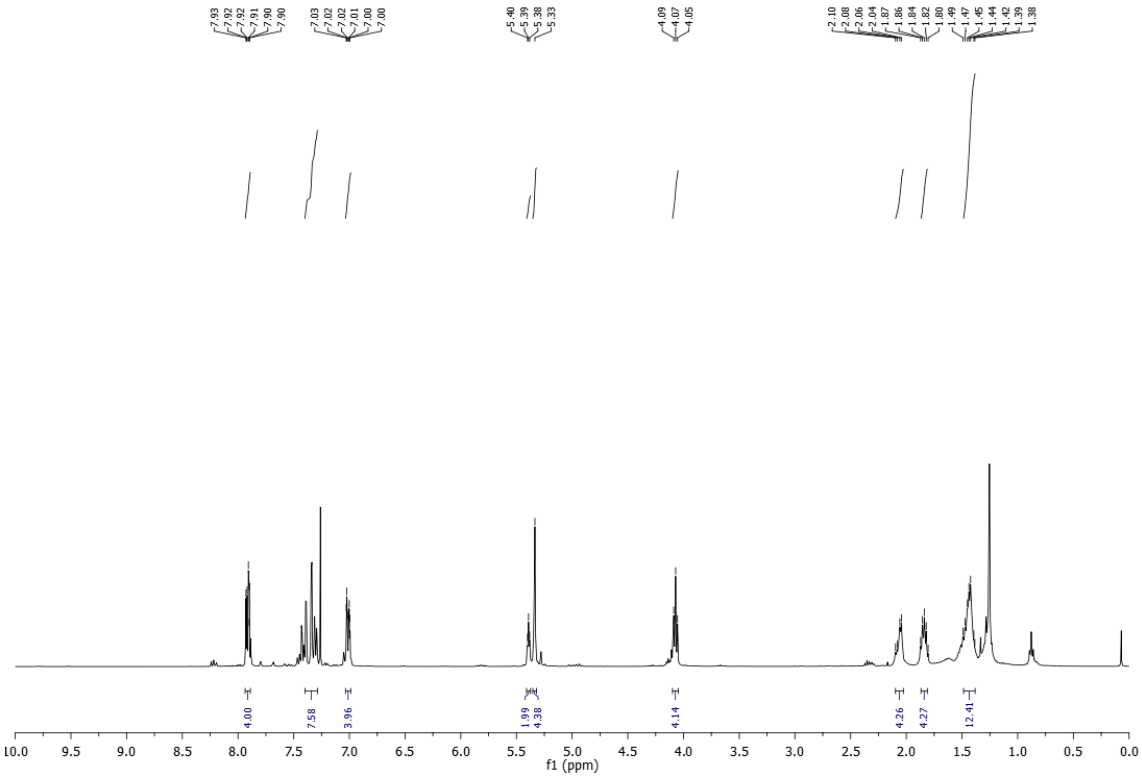

Supplementary Figure 2. ^{1}H-NMR spectrum of **mac-AQ** ($CDCl_3$, 298 K)

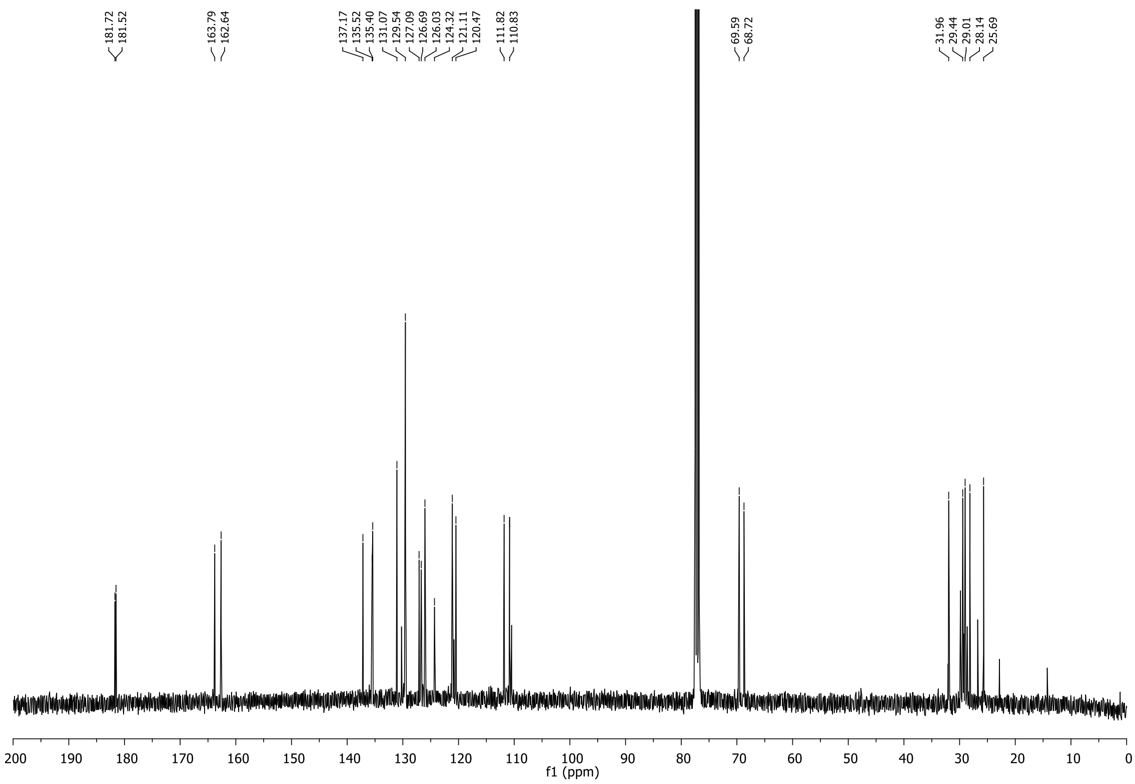

Supplementary Figure 3. ^{13}C-NMR spectrum of **mac-AQ** ($CDCl_3$, 298 K)

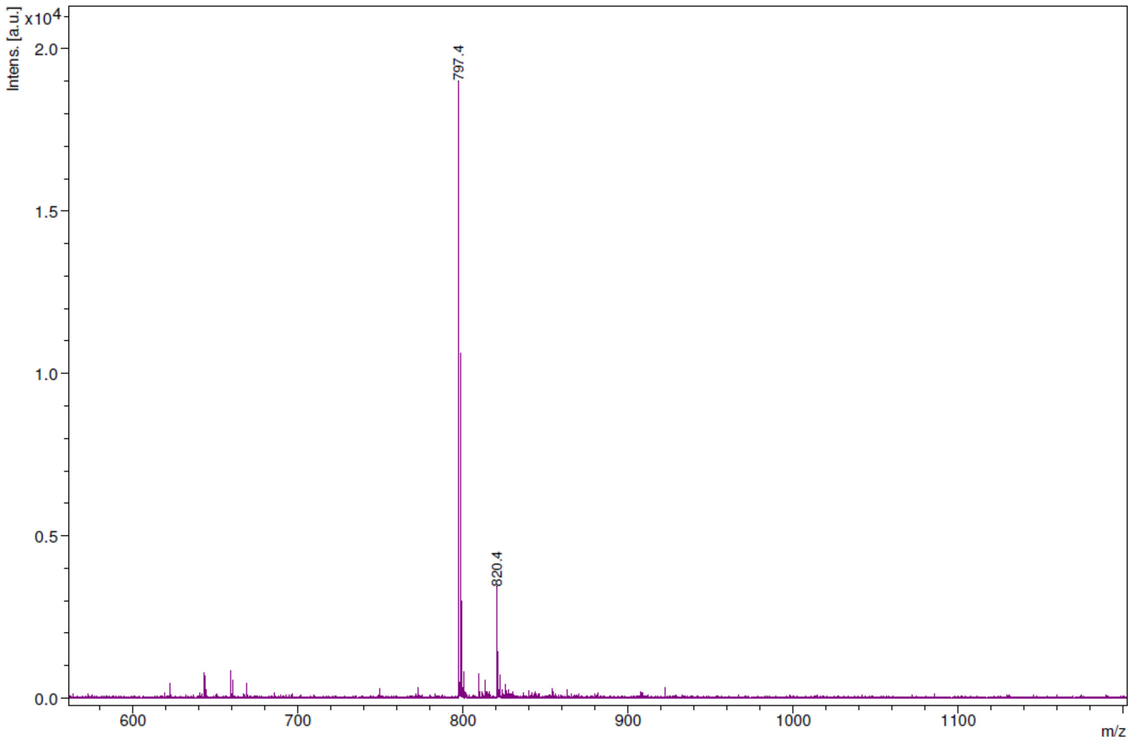

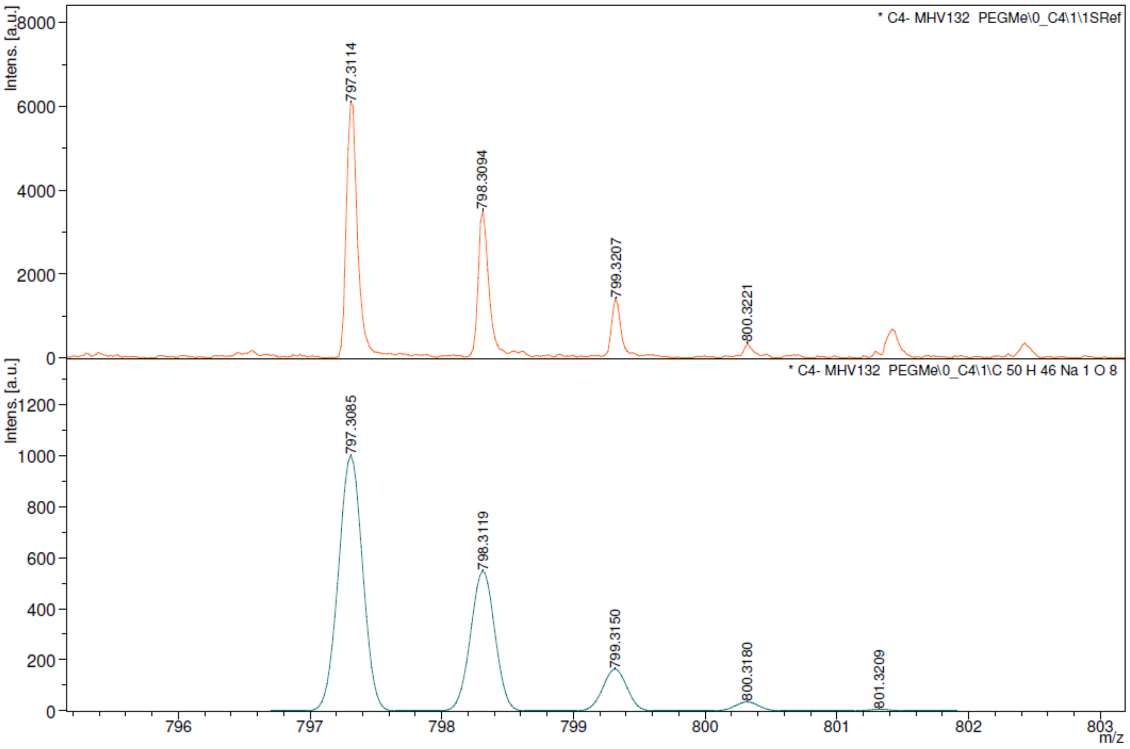

Supplementary Figure 4. MALDI-TOF spectra of **mac-AQ**.

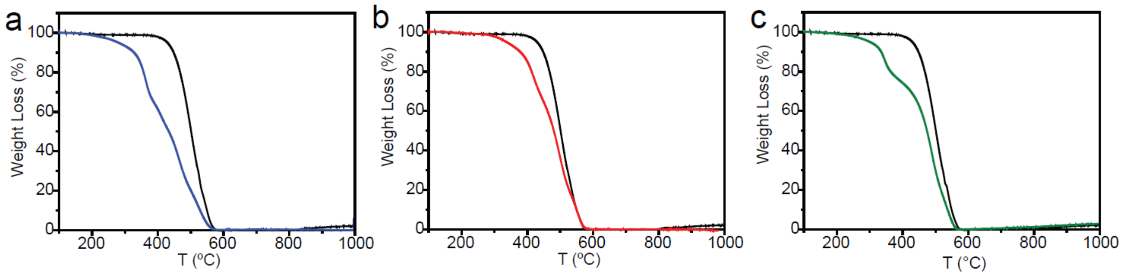

Supplementary Figure 5. TGA plots (ramp of 10 °C min^{-1} under air from 100 to 1000 °C) of a) **MINT-exTTF** (blue), b) **MINT-pyr** (red) and c) **MINT-AQ** (green) compared with (6,5)-SWNT (black).

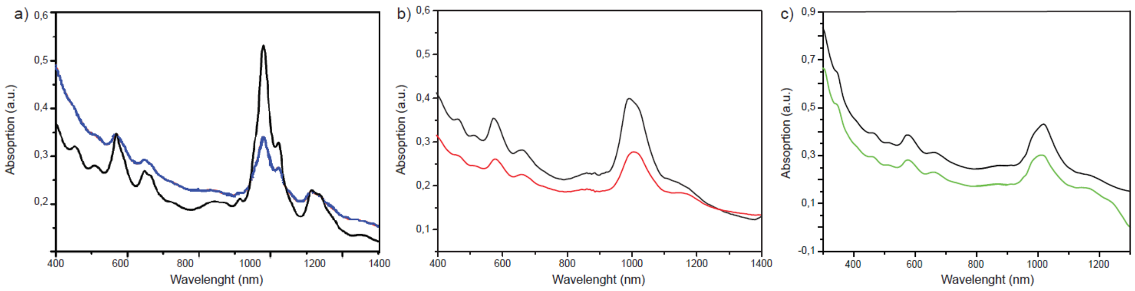

Supplementary Figure 6. UV-Vis spectra (D_2O / SDS (1 wt%) at room temperature) of a) **MINT-exTTF** (blue), b) **MINT-pyr** (red) and c) **MINT-AQ** (green) compared with (6,5)-SWNT (black).

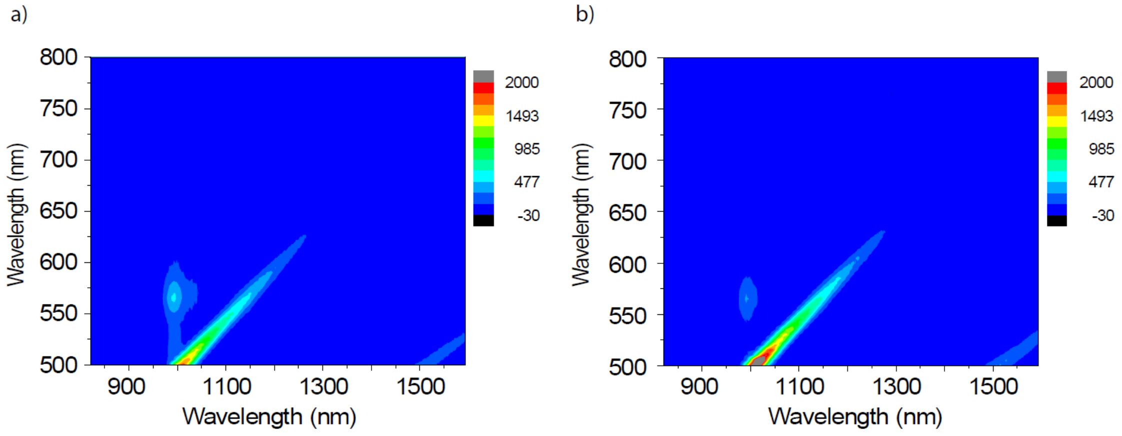

Supplementary Figure 7. PLE map of a) (6,5)-SWNT and b) **MINT-AQ**

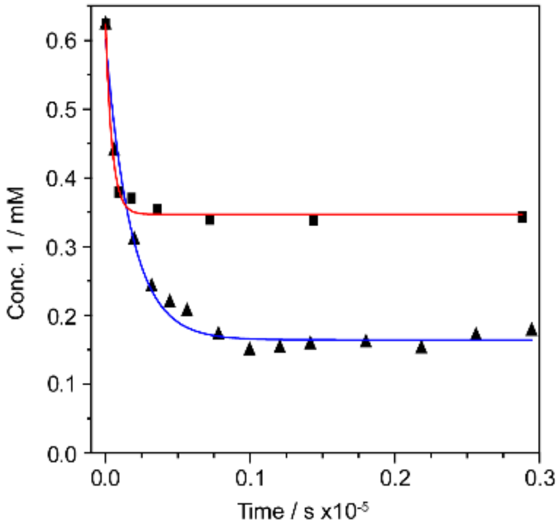

Supplementary Figure 8. Data from kinetic experiment of **MINT-AQ** (squares) and RCM templated with C_{60} (triangles). The fit to a mono-exponential decay are shown in red and blue respectively.

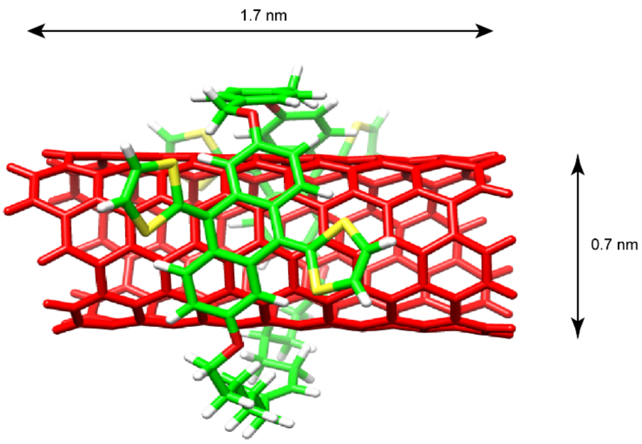

Supplementary Figure 9. Optimized structure of **MINT-exTTF**. Dimension of the modelled carbon nanotubes are indicated in the figure.

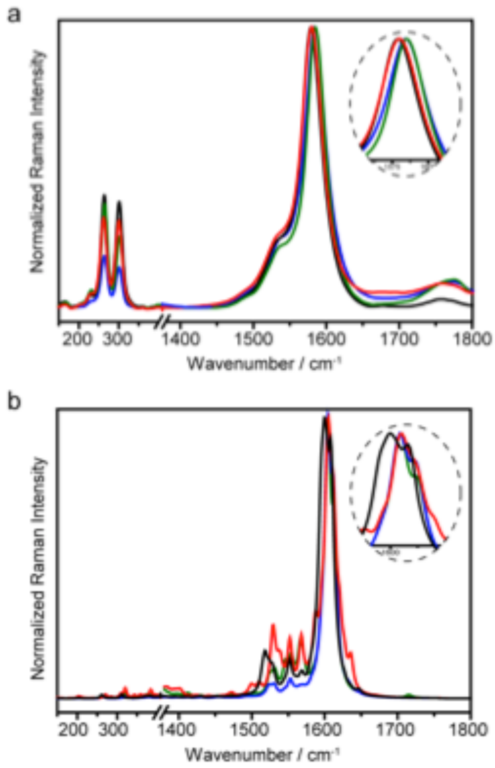

Supplementary Figure 10. a) Experimental Raman spectra of SWNT (black), **MINT-exTTF** (blue), **MINT-pyr** (red) and **MINT-AQ** (green). b) Calculated Raman spectra at CAM-B3LYP/3-21g* level of theory. Same color code.

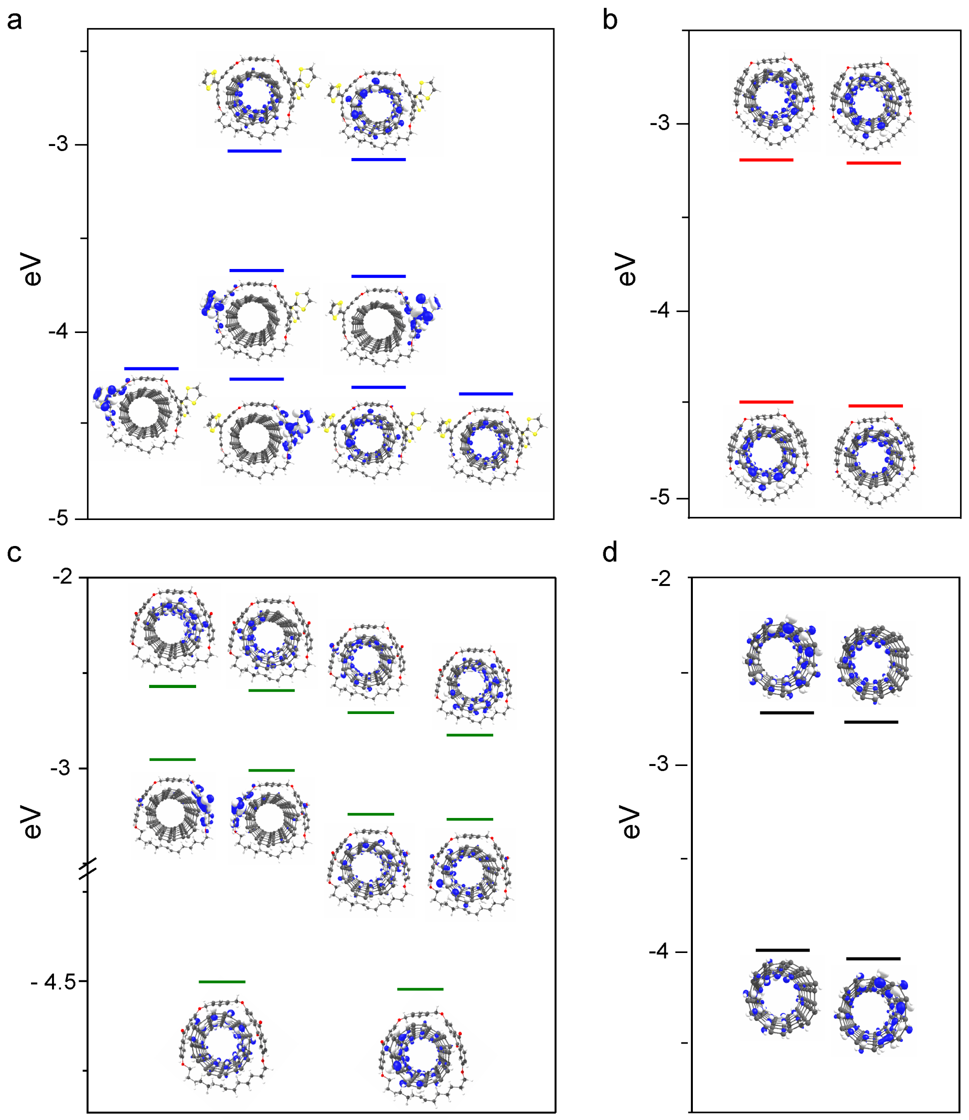

Supplementary Figure 11. Energy diagrams and topologies of the frontier molecular orbitals of, a) **MINT-exTTF (blue),** b) **-pyr (red)**, c) **-AQ (green) and** d) **(6,5)-SWNT (black)** calculated at B97D/3-21G* level of theory.

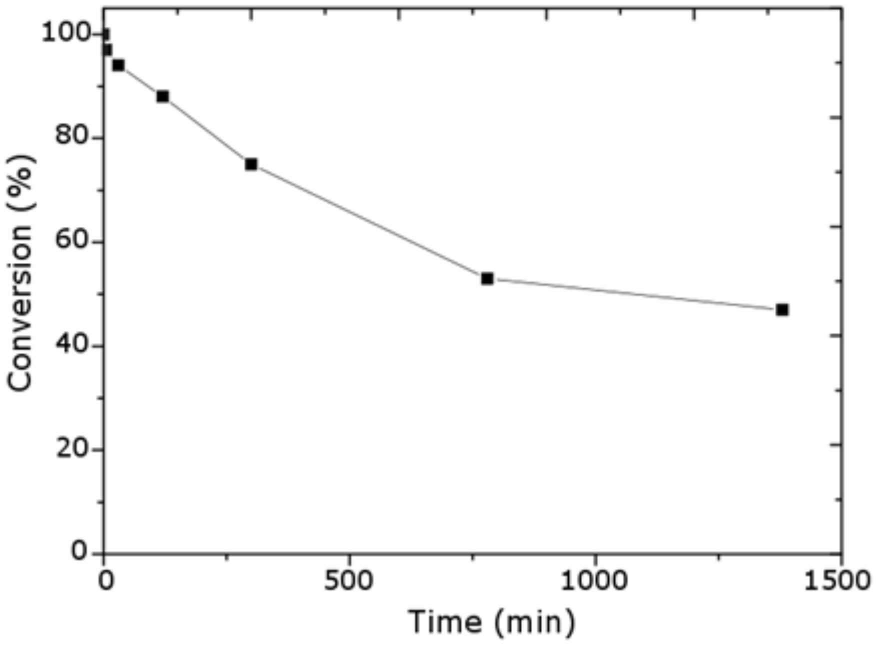

Supplementary Figure 12. Reduction of 1-nitronaphtalene without catalyst.

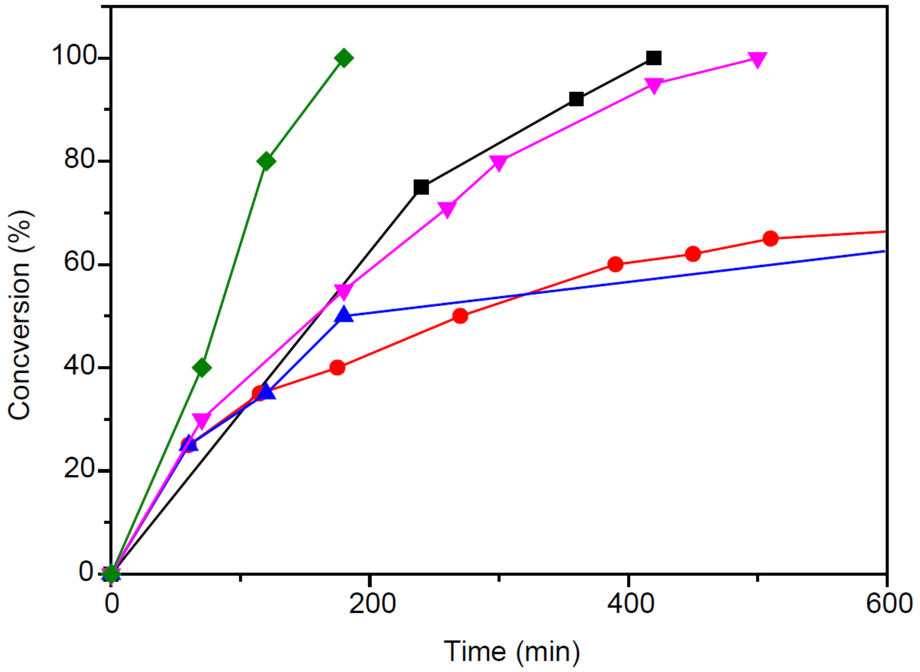

Supplementary Figure 13. Solvent optimization on the reduction of 1-nitronaphtalene catalyzed by **(6,5)-SWNT** (green: DMSO; black: Ethanol; purple: Methanol; blue: H_2O; red: $CHCl_3$).

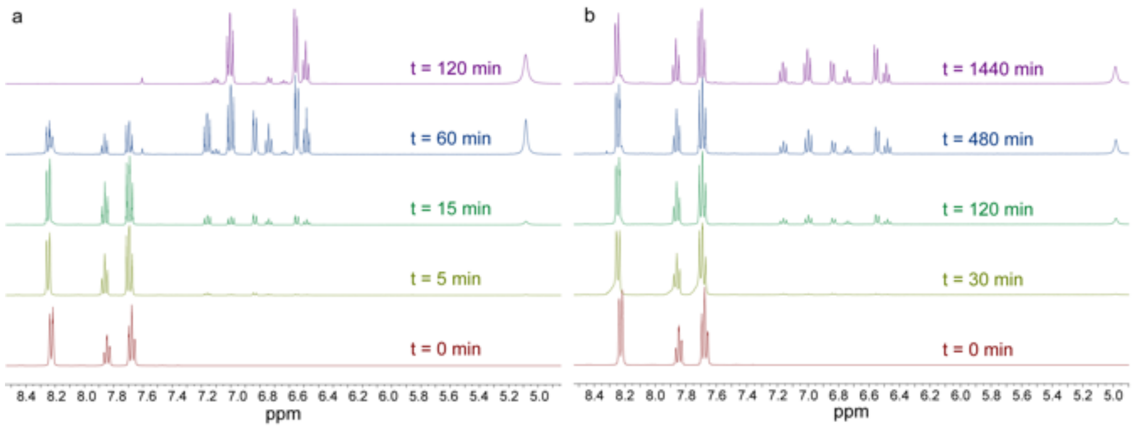

Supplementary Figure 14. Partial ^{1}H-NMR (400 MHz, d_6-DMSO) spectra of the reduction of Ph-NO_2 a) catalyzed by sample **MINT-exTTF** and b) without catalyst.

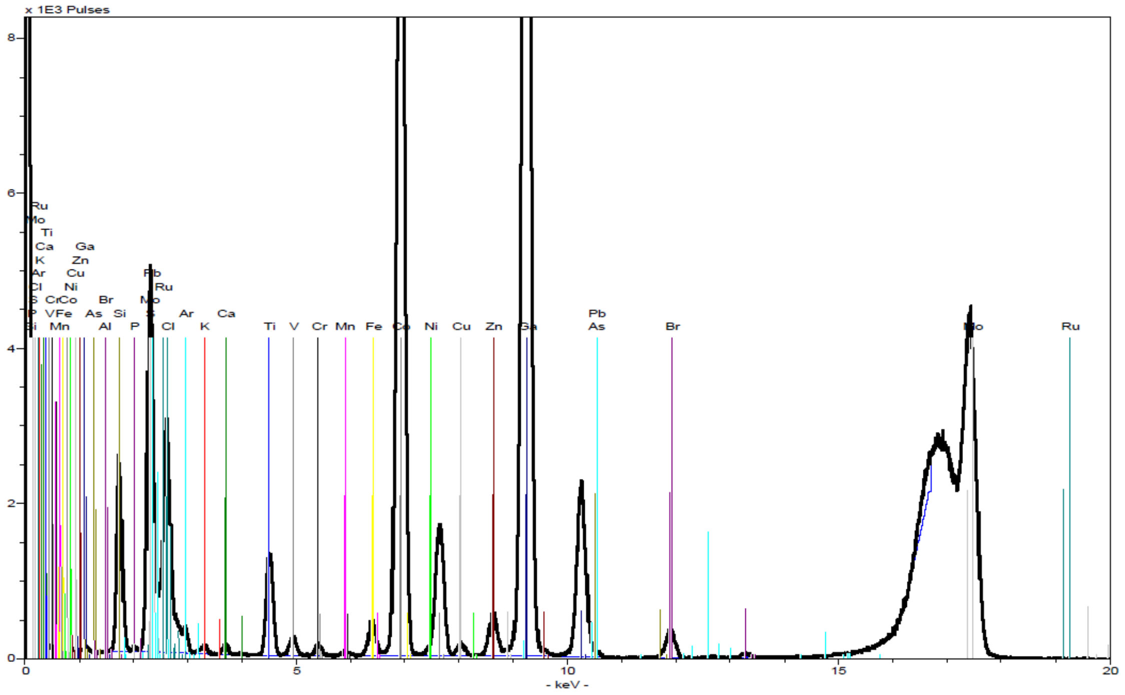

Supplementary Figure 15. FTRX spectrum of **MINT-exTTF** sample.

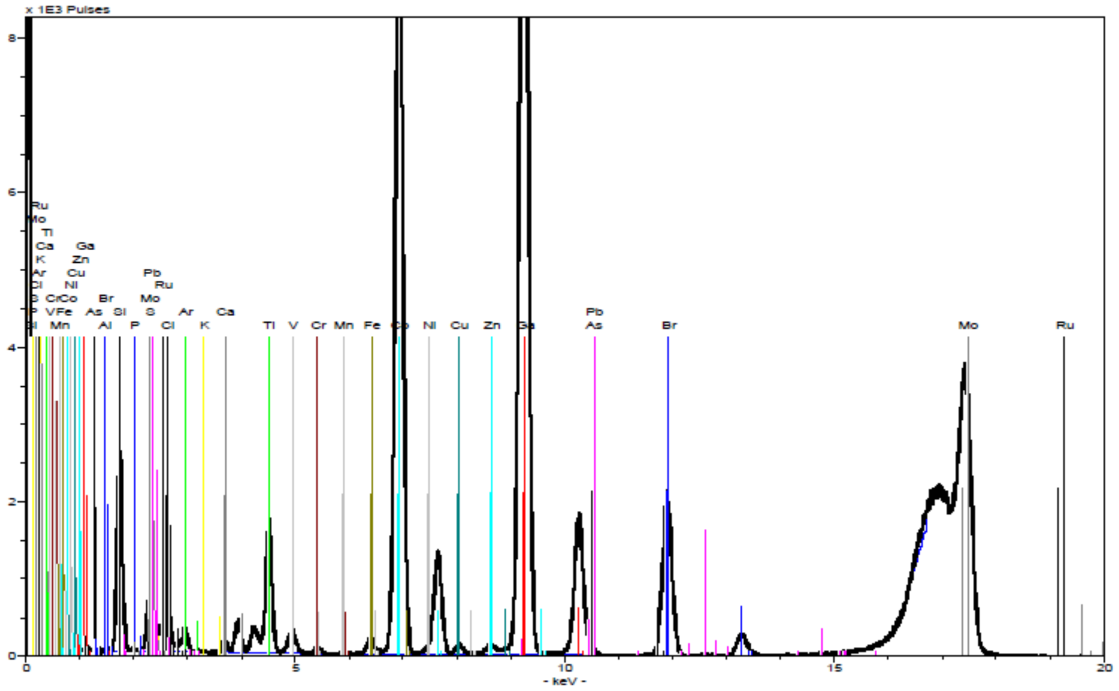

Supplementary Figure 16. FTRX spectrum of **MINT-pyr** sample.

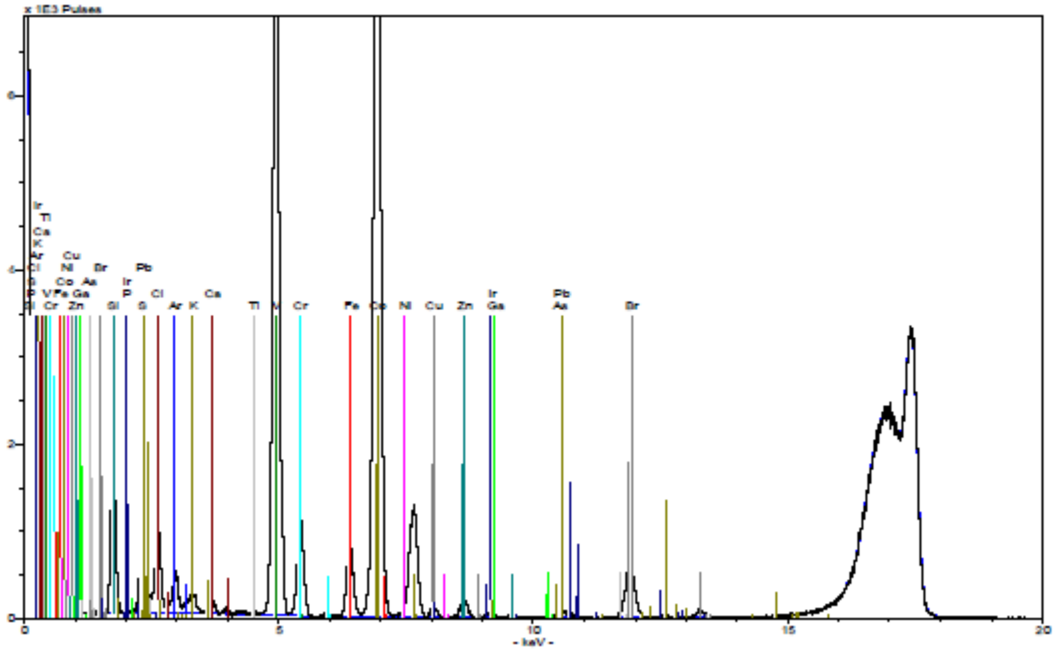

Supplementary Figure 17. FTRX spectrum of **MINT-AQ** sample.

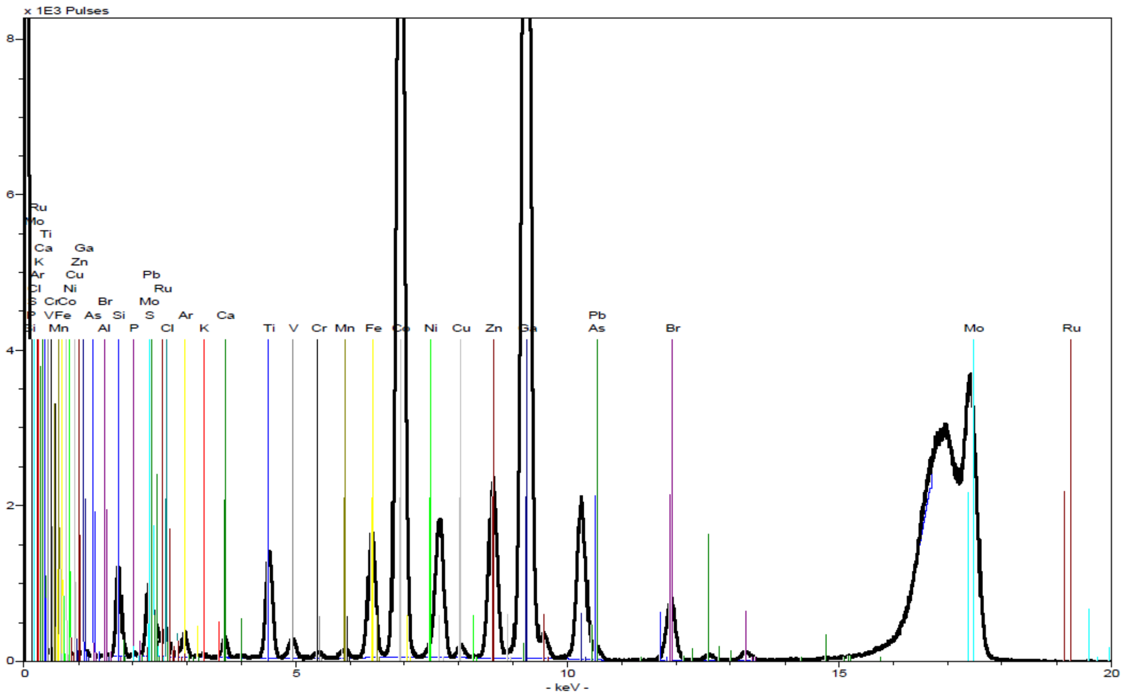

Supplementary Figure 18. FTRX spectrum of **(6,5)-SWNT** sample.

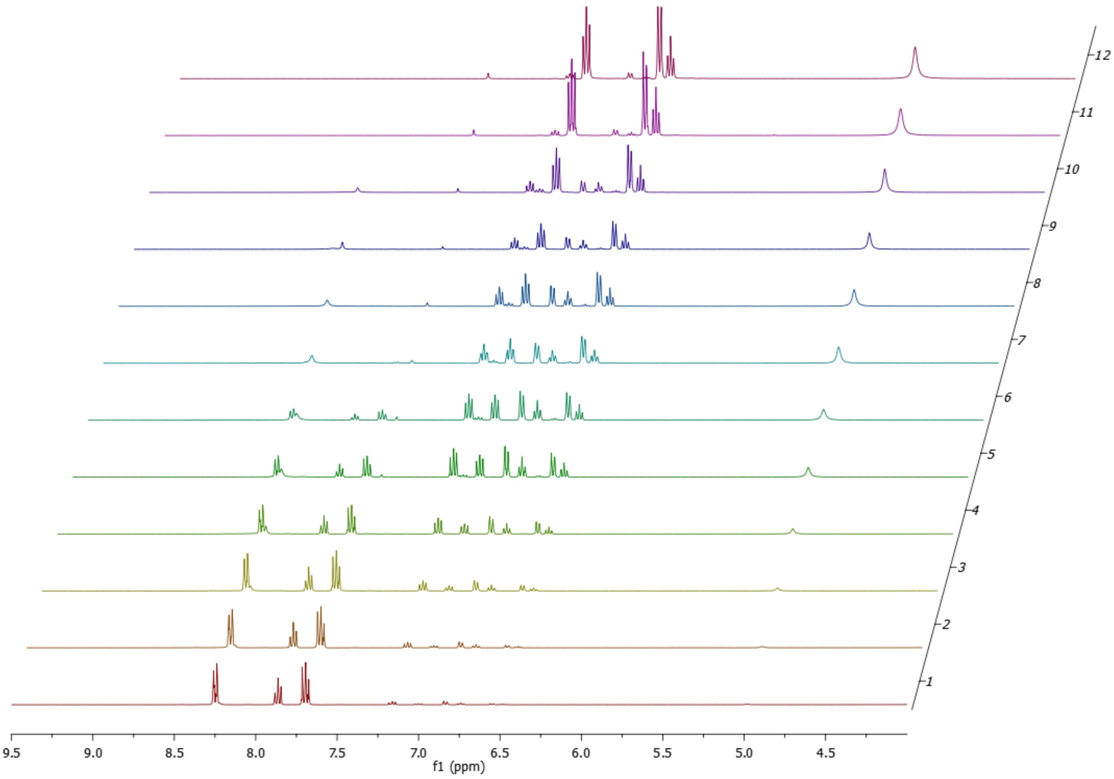

Supplementary Figure 19. Example of NMR kinetic profiles. Nitrobenzene reduction catalyzed by catalyst **MINT-exTTF.**

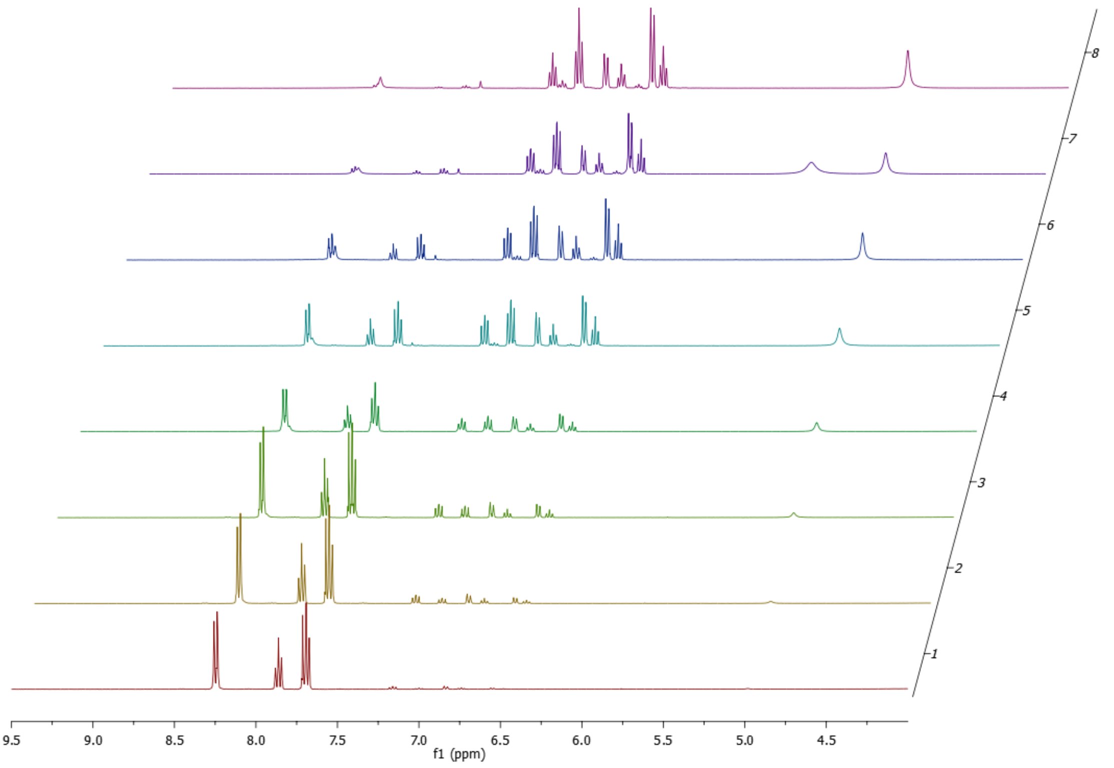

Supplementary Figure 20. Example of NMR kinetic profiles. Nitrobenzene reduction catalyzed by catalyst **MINT-pyr.**

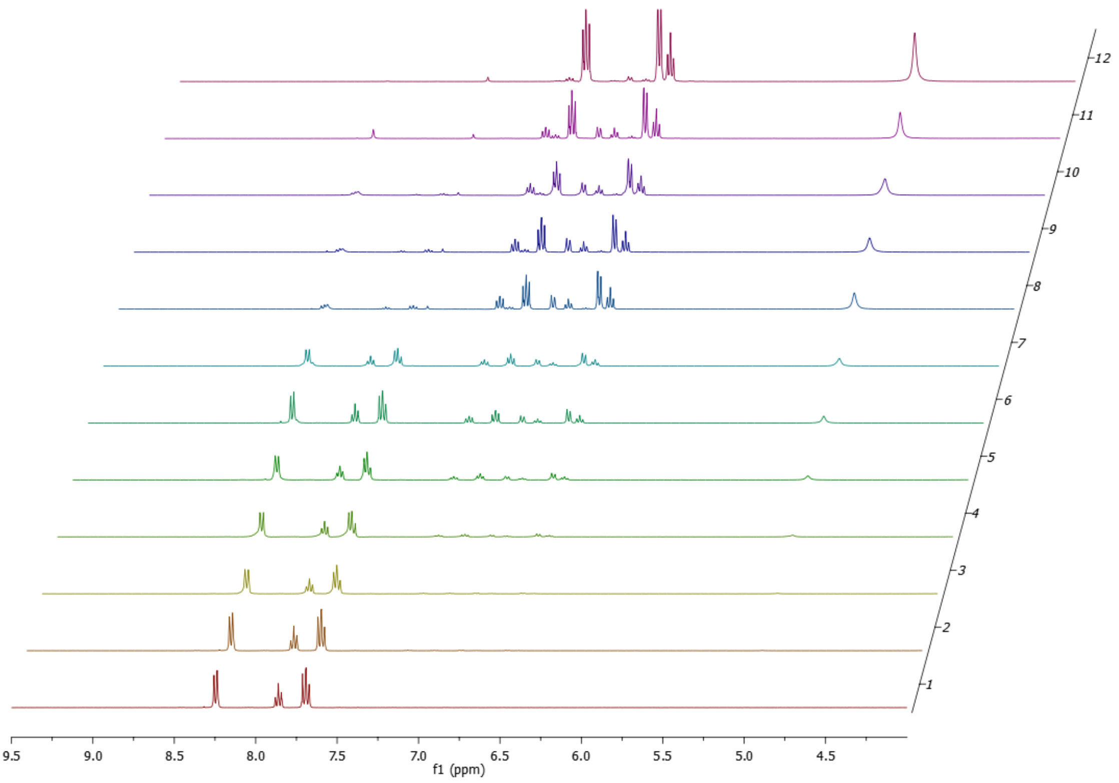

Supplementary Figure 21. Example of NMR kinetic profiles. Nitrobenzene reduction catalyzed by catalyst **MINT-AQ.**

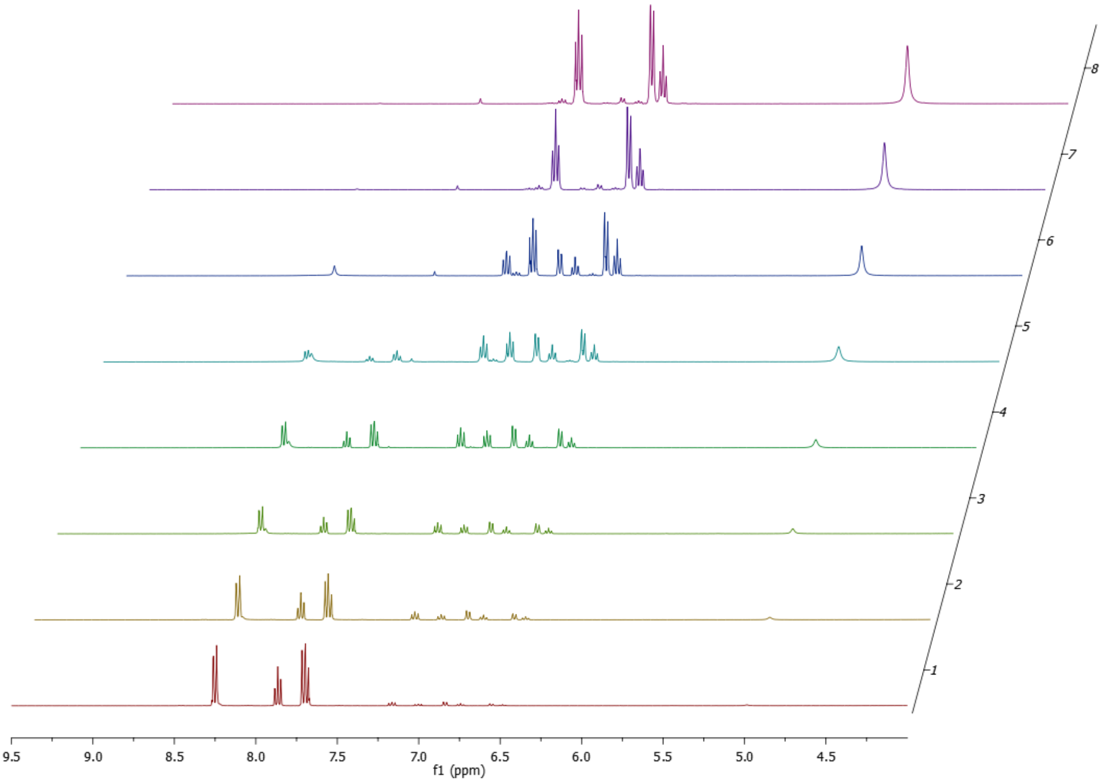

Supplementary Figure 22. Example of NMR kinetic profiles. Nitrobenzene reduction catalyzed by catalyst **(6,5)-SWNT.**

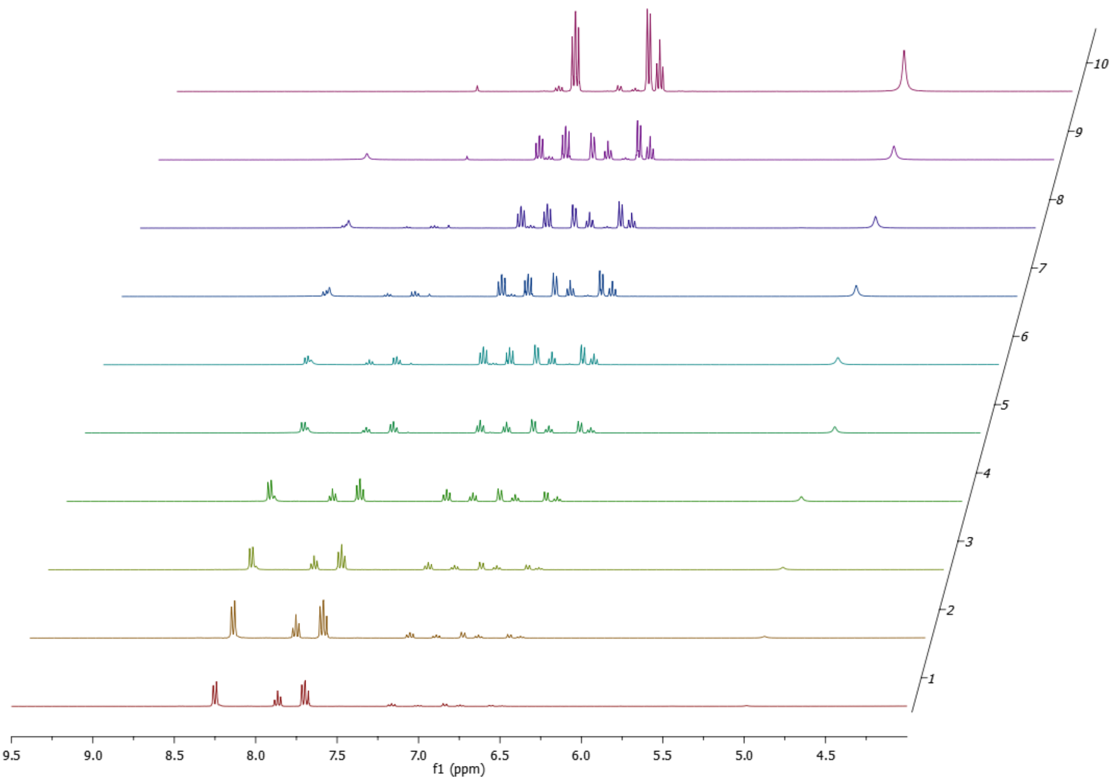

Supplementary Figure 23. Nitrobenzene reduction catalyzed by catalyst **SWNT-exTTF.**

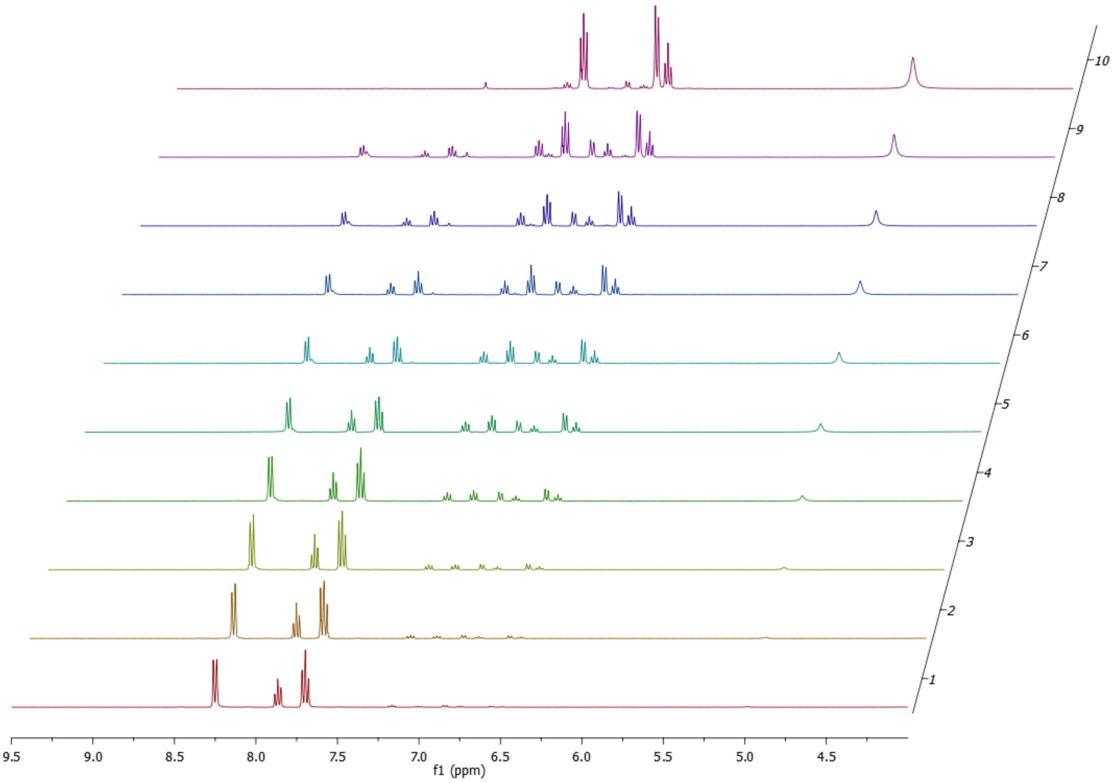

Supplementary Figure 24. Nitrobenzene reduction catalyzed by catalyst **SWNT-pyr.**

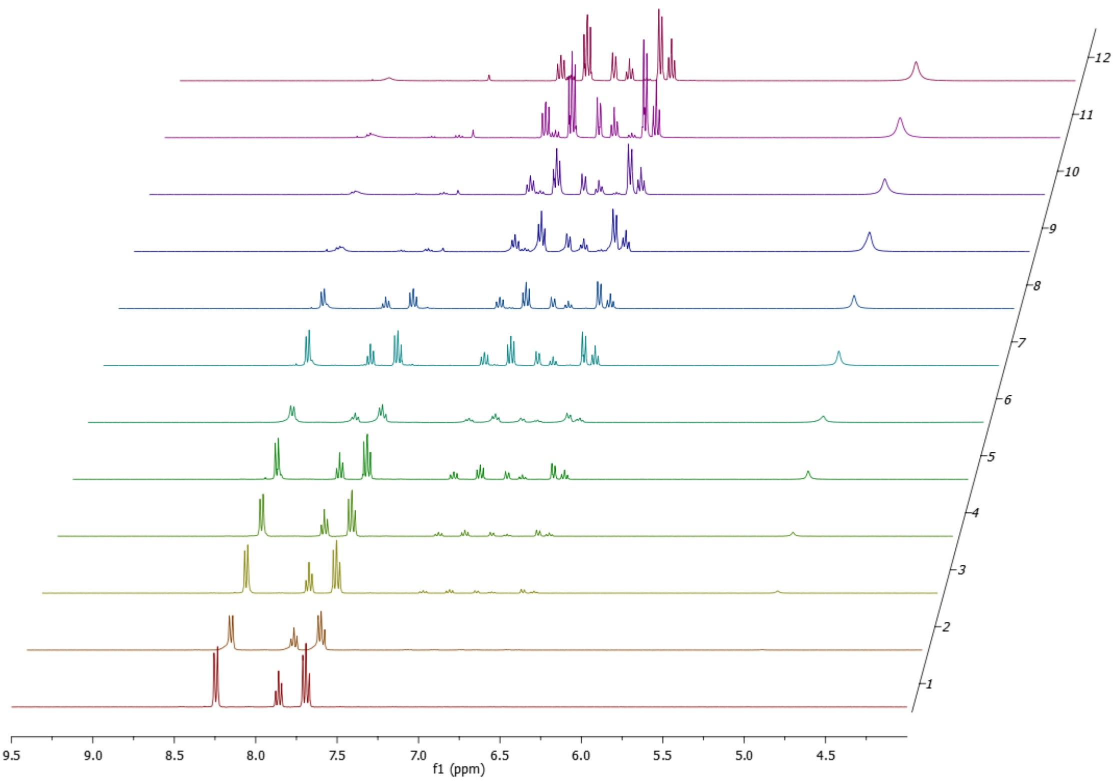

Supplementary Figure 25. Nitrobenzene reduction catalyzed by catalyst **SWNT-AQ.**

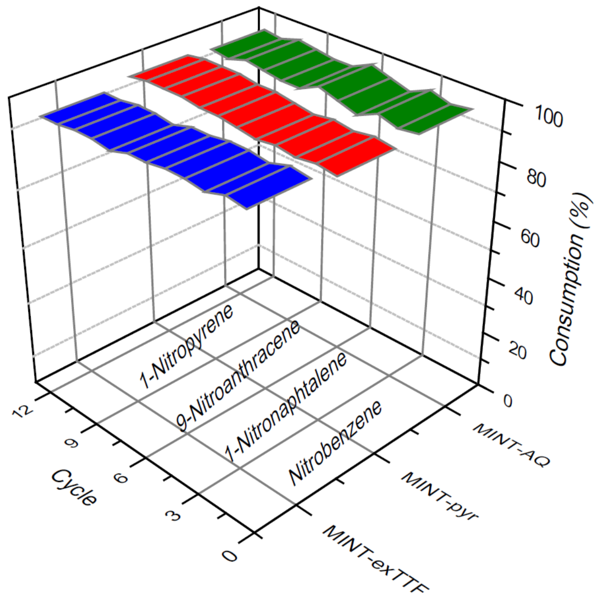

Supplementary Figure 26. Recycling studies of the **MINT-exTTF** (blue), **MINT-pyr** (red) and **MINT-AQ** (green) catalysts.

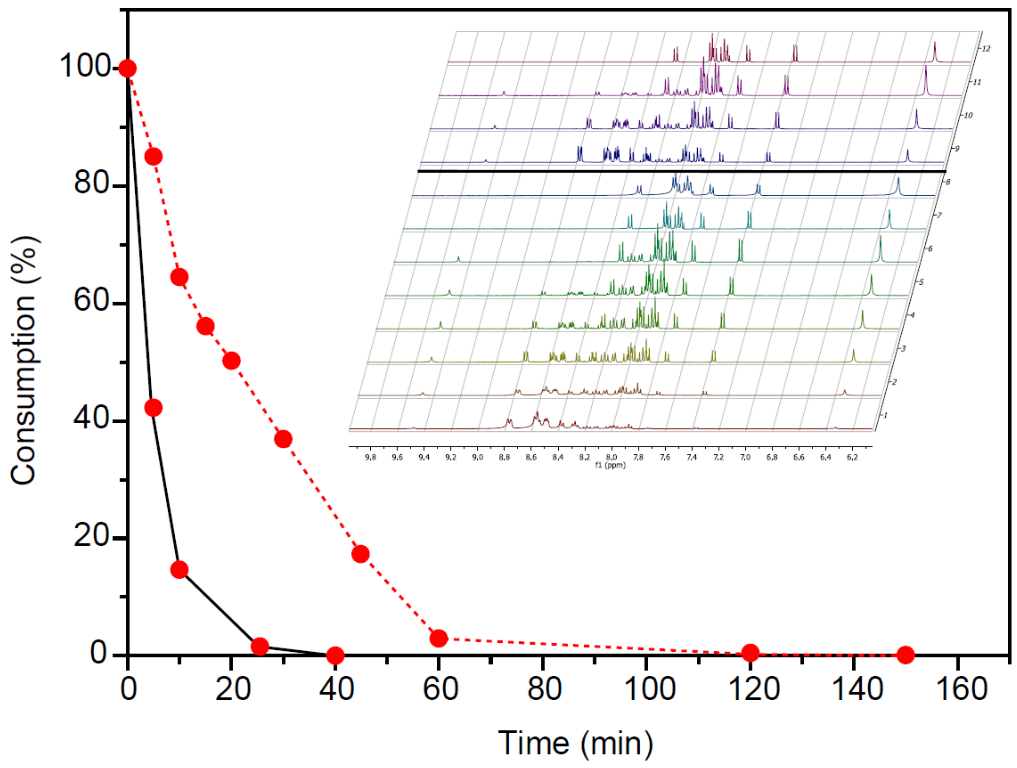

Supplementary Figure 27. Recycling study of the reduction of 1-nitropyrene catalyzed by **SWNT-pyr** (red dots) on the first cycle (solid black line) and on the second cycle (red dashed line). Inset: NMR kinetic profile of the first and second cycle.

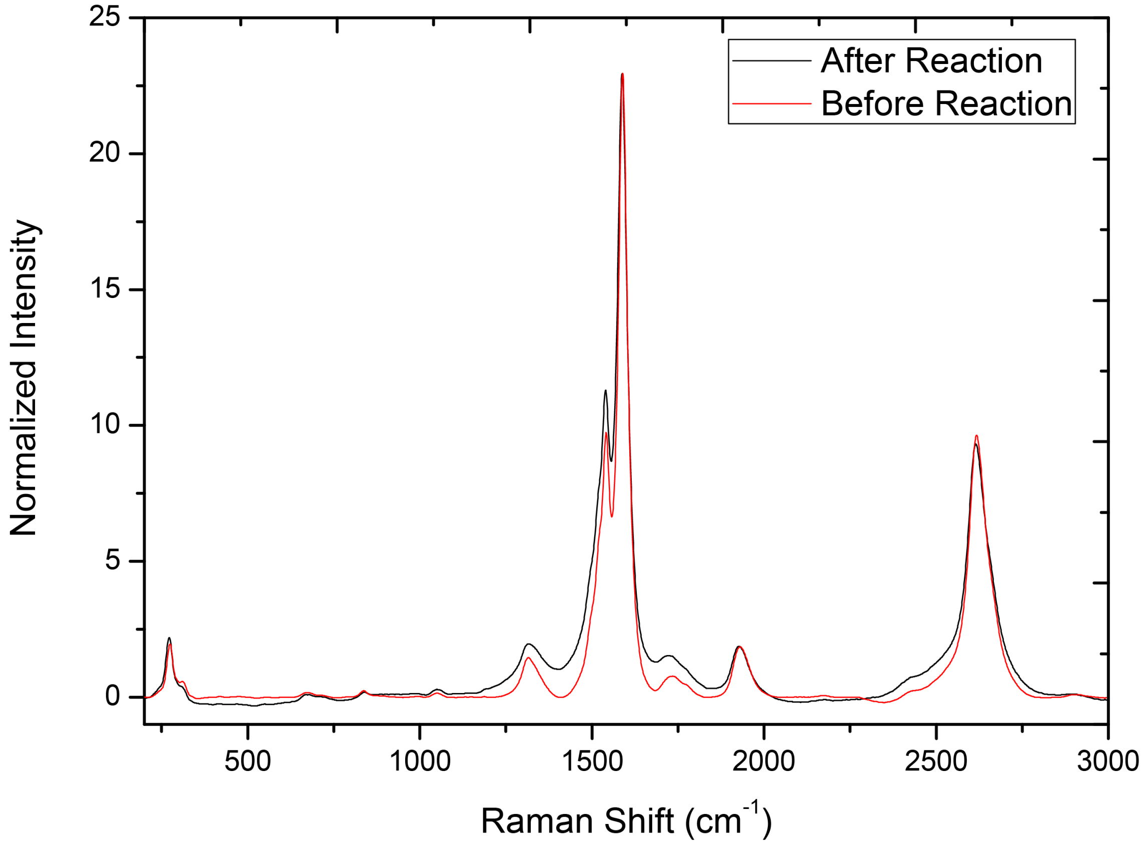

Supplementary Figure 28. Raman spectra of **MINT-AQ** before and after the reduction reaction, showing its structural integrity.

Supplementary Tables

Supplementary Table 1. Energy parameters (kcal mol^{-1}) of the interaction between macrocyles and guest SWNTs at the B97-D/3-21G* level.

	E_{int}	E_{def}	$E_{bind} = E_{int} + E_{def}$
MINT-AQ	-97.74	22.81	-74.93
MINT-pyr	-108.61	40.74	-67.88
MINT-exTTF	-103.58	12.01	-91.57

Supplementary Table 2. Energy parameters (kcal mol^{-1}) of the interaction between the nitroaromatic molecules and the (6,5)-SWNT at the B97-D/3-21G* level.

	E_{int}	E_{def}	$E_{bind} = E_{int} + E_{def}$
Nitrobenzene	-16.05	0.59	-15.45

1-Nitronaphtalene	-22.22	0.49	-21.73
9-Nitroanthracene	-28.06	1.52	-26.54
1-Nitropyrene	-32.03	0.65	-31.38

Supplementary Table 3. Energy parameters (kcal mol^{-1}) of the interaction between the nitroaromatic molecules and the MINT-AQ at the B97-D/3-21G* level.

	E_{int}	E_{def}	$E_{bind} = E_{int} + E_{def}$
Nitrobenzene	-16.11	0.30	-15.81
1-Nitronaphtalene	-22.66	0.61	-22.04
9-Nitroanthracene	-28.56	1.48	-27.08
1-Nitropyrene	-33.09	1.07	-32.02

Supplementary Table 4. Calculated charge transfer from macrocycle to nanotube.

	Charge transfer [e]
MINT-AQ (3-21g*)	+0.011
MINT-AQ (6-31g*)	+0.087
MINT-pyr	+0.005
MINT-exTTF	-0.043

Supplementary Table 5. Metal analysis by TRXF

Element	**MINT-exTTF**[a]	**MINT-pyr**[a]	**MINT-AQ**[a]	**6,5-SWNT**[a]
Al	-	-	-	-
Si	146.72	191.56	129.9	78.06
P	-	-	0.44	-
S	70.26	2.125	10.12	2.084
Cl	28.06	11.879	12.906	4.055
K	0.509	-	1.214	0.142
Ca	0.534	2.488	0.894	1.119
Ti	3.233	5.763	0.1	3.713
V	0.111	0.172	0.124	0.139
Cr	0.200	0.145	0.011	0.122
Mn	0.040	-	-	0.153
Fe	0.505	0.289	1.079	2.053
Co	13.435	12.748	11.285	15.438
Ni	0.032	0.023	0.015	0.063
Cu	0.117	0.101	0.071	0.127
Zn	0.340	0.084	0.147	1.620
Ga[b]	10.000	10.000	10.000	10.000

As	0.027	0.028	0.035	0.049
Br	0.168	1.185	0.315	0.396
Mo	17.82	25.69	20.48	29.68
Ru	17.02	0.494	0.941	-
Pb	0.017	0.006	-	0.086

[a]Concentrations in mg L^{-1}, [b]Internal standard

Supplementary Equations

Supplementary Equation 1.

$$E_{int}(AB) = E_{AB}^{\alpha\beta} - E_{A}^{\alpha\beta} - E_{B}^{\alpha\beta}$$

Supplementary Equation 2.

$$E_{bind} = E_{def} + E_{int}$$

Supplementary References

1 Canevet, D. *et al.* Macrocyclic Hosts for Fullerenes: Extreme Changes in Binding Abilities with Small Structural Variations. *J. Am. Chem. So.* **133**, 3184-3190, doi:10.1021/ja111072j (2011).

2 de Juan, A. *et al.* Mechanically Interlocked Single-Wall Carbon Nanotubes. *Angew. Chem. Int. Ed.* **53**, 5394-5400, doi:10.1002/anie.201402258 (2014).

3 Lopez-Moreno, A. & Perez, E. M. Pyrene-based mechanically interlocked SWNTs. *Chem. Commun.* **51**, 5421-5424, doi:10.1039/C4CC08970G (2015).

4 Gaussian 09 Revision C.01 (Gaussian, Inc., Wallingford, CT, USA, 2009).

5 Grimme, S. Semiempirical GGA-type density functional constructed with a long-range dispersion correction. *J. Comp. Chem.* **27**, 1787-1799, (2006).

6 Yanai, T., Tew, D. P. & Handy, N. C. A new hybrid exchange–correlation functional using the Coulomb-attenuating method (CAM-B3LYP). *Chem. Phys. Lett.* **393**, 51-57, (2004).

7 Binkley, J. S., Pople, J. A. & Hehre, W. J. Self-consistent molecular orbital methods. 21. Small split-valence basis sets for first-row elements. *J. Am. Chem. Soc.* **102**, 939-947, (1980).

8 Boys, S. F. & Bernardi, F. The calculation of small molecular interactions by the differences of separate total energies. Some procedures with reduced errors. *Mol. Phys.* **19**, 553-566, (1970).

9 Gao, Y., Ma, D., Wang, C., Guan, J. & Bao, X. Reduced graphene oxide as a catalyst for hydrogenation of nitrobenzene at room temperature. *Chem. Commun.* **47**, 2432-2434, (2011).

10 Leret, S. *et al.* Bimodal supramolecular functionalization of carbon nanotubes triggered by covalent bond formation. *Chem. Sci.* **8**, 1927-1935, (2017).

11 Martinez-Perinan, E. *et al.* The mechanical bond on carbon nanotubes: diameter-selective functionalization and effects on physical properties. *Nanoscale* **8**, 9254-9264, (2016).

12 Rao, C. N. R. & Voggu, R. Charge-transfer with graphene and nanotubes. *Mater. Today* **13**, 34-40, (2010).
13 Bachilo, S. M. *et al.* Structure-Assigned Optical Spectra of Single-Walled Carbon Nanotubes. *Science* **298**, 2361-2366, (2002).
